# The Effectiveness of Massage and Warm Compresses on Perineal Trauma, Hemorrhage, Length of Episiotomy and Pain: A Randomized Controlled Trial

**DOI:** 10.1055/a-2730-1313

**Published:** 2025-11-19

**Authors:** Gamze Acavut, Gülten Güvenç, Kazım Emre Karaşahin

**Affiliations:** 1 566936 Midwifery Department, Ankara Medipol University, Ankara, Turkey; 2 531765 Gulhane Faculty of Nursing, University of Health Sciences, Ankara, Turkey; 3 574983 Obstetrics, University of Health Sciences Gulhane Health Sciences Institute, Ankara, Turkey

**Keywords:** perineal trauma, perineal massage, hemorrhage, episiotomy, pain

## Abstract

**Background:**

There are limited number of randomized controlled studies on the effectiveness of perineal massage and warm compresses in reducing perineal trauma.

**Aim:**

To evaluate the effectiveness of massage and warm compresses implemented by nurses and midwives on perineal trauma, volume of hemorrhage, length of episiotomy, and pain.

**Methods:**

The single-center, single-blind randomized controlled trial (RCT) included 120 pregnant women in labor. Women were randomly divided into four groups: receiving massage only, warm compress only, both massage and warm compress, and the control group.

**Results:**

The application of warm compresses and massage was effective for reducing perineal trauma (p<0.001). Warm compress application was found to be effective to decrease first-degree trauma (p<0.001). The hemorrhage volume and length of episiotomy for the intervention groups were lower than in the control group (p<.001). Massage and warm compress interventions were effective for reducing pain (p<0.05).

**Conclusions:**

Massage and warm compress methods are effective in reducing trauma, pain, hemorrhage, and episiotomy length. Furthermore, the use of the two methods together does not provide an advantage.

## Introduction

Perineal trauma is a type of damage that is the result of a tear or laceration during childbirth 1 2 3 . Trauma can cause an unwanted physical and psychological health burden. Perineal pain, hemorrhage, prolapse, urinary incontinence, sexual dysfunction, and anxiety in later periods are some of the morbidities 2 4 . The problems that occur due to perineal trauma can negatively affect the quality of life of women 2 5 .

Maternal age, macrosomia, and the episiotomy method are stated as risk factors in perineal traumas 2 6 . Studies show that 85.0% of women are exposed to birth-related perineal trauma and 59.0–73.0% are exposed to some type of laceration 7 8 . It is reported that most traumas are related to the application of an episiotomy 5 9 10 . An episiotomy, which is used to facilitate delivery and shorten the second stage of labor, can lead to perineal trauma and complications. Studies have shown that an episiotomy is frequently performed on primiparous women and that they are exposed to trauma 2 7 11 .

Since perineal trauma is a condition with serious complications in early and later stages, prevention is easier and more beneficial and economical than treatment. For this reason, some methods are used to protect the perineum in order to reduce perineal trauma, manage pain, and reduce hemorrhage 12 13 14 .

It is stated that perineal massage and warm compresses increase vasodilation and perineum flexibility and decrease the episiotomy rate and interventional vaginal delivery 5 12 13 14 . Studies have shown that these methods, which can be applied in the first and second stages of labor, are effective in reducing perineal trauma, hemorrhage, and pain 5 12 15 16 . It is therefore recommended that midwives and nurses use such methods that reduce pain and trauma and that can be applied easily 17 18 . Although there are studies on this subject, some of studies evaluated only these methods alone, and in some of them, only parameters affected by pain, trauma, and episiotomy were evaluated alone 12 13 14 15 16 . In this study, methods were used both individually and together, and it was aimed to provide evidence for the literature by evaluating their effects on more than one parameter.

The aim of this randomized control study was to evaluate the impact that massage and warm compresses applied by midwives and nurses during the first and second stages of delivery on trauma, pain around the perineum, hemorrhage during labor, and the length of episiotomy in a sample of primiparous women.

## Methods

### Design

A single-blind, single-center randomized controlled trial (RCT) design was used to compare four groups (massage, warm compress, massage and warm compress, control) of participants. This study was conducted in a training and research hospital in Ankara, Türkiye. CONSORT 2010 guidelines for reporting RCTs were followed to describe the methods. The study was registered on ClinicalTrials.gov (NCT06005077).

### Participants

This study involved primiparous women in gestational week 38 and over, with a singleton pregnancy and the fetus in the vertex position. The exclusion criteria were previous uterine surgery, a chronic/hematological disease, or a fetal abnormality. In addition, patients for whom vacuum or forceps were applied during delivery were excluded.

### Sample selection and randomization

The study’s sample population included 716 women who gave birth vaginally. The sample size was determined using the G*Power program. While calculating sample size, data of the study in which trauma was evaluated by applying perineal massage and a warm compress during labor were taken into account. The effect size was found to be 0.522 15 . It was planned to conduct the study with a total of 120 participants at a 95.0% confidence interval and 95.0% power. Randomization was carried out by a researcher using a program through RANDOM.ORG. This study was conducted as a single-blind study in which women did not know whether they were in the intervention or control group. To prevent bias, a second researcher did not assign the groups and did not perform outcome assessments.

A total of 120 pregnant women were included in the study, with 30 assigned to the massage (PM) group, 30 to the warm compress (PWC) group, 30 to the massage and warm compress (PM+PWC) group, and 30 to the non-intervention group, which received routine midwife/nurse care. Assessed for eligibility were 716 participants, and 574 participants were excluded because of multiparity, gestational age, chronic illness, or unwillingness to participate. A total of 142 women were randomized. In each group, 30 participants were analyzed, and 22 of them were excluded because of cesarean delivery and vacuum implementation.

### Data collection and intervention

Data collection was performed between May 2018 and September 2019. All participants provided informed consent before participation. The control group was given routine care by nurses and midwives. The participants were continuously monitored until their transfer to a postpartum clinic. A face-to-face interview was conducted throughout the research.

Massage only: Massage was performed with the fingers with the woman in the lithotomy position. U-shaped movements were applied to 3–4 cm of the inner part of the vagina, from the sidewalls and towards the rectum. This procedure was carried out for 10 minutes in three phases, based on the cervical dilation process. It was implemented when the cervix was dilated to 3–4 cm, 5–7 cm, or 8–10 cm.

Warm compress only: A compress moistened with 40°C water was placed on the perineal area. The compress was replaced with a new one when it became cold or dirty. This procedure was performed for 30 minutes based on the progress of cervical dilatation. It was performed when dilatation of the cervix was 3–4 cm, 5–7 cm or 8–10 cm.

Both massage and warm compress: When dilation of the cervix reached 3–4 cm, a warm compress was first applied for 30 minutes. Massage was then performed for 10 minutes. Both techniques were repeated when cervical dilation was 5–7 cm or 8–10 cm.

## Pilot study

Prior to the study, massage and warm compresses were applied to 12 pregnant women to test the procedure. The pilot study was performed via data collection forms and a hemorrhage follow-up/collector bag. As a result of the preliminary application, application steps and data collection forms were evaluated and then necessary revisions made. The findings of the pilot assessment were not included in main study findings.

## Measurement tools

### The sociodemographic and obstetric data collection form

These data were used to assess the sociodemographic and obstetric characteristics of the participants. This form, prepared by researchers, includes 15 questions about the participant’s age, educational status, week of gestation, and health problems experienced during pregnancy 1 4 5 8 11 16 17 18 .

### The stage of labor and newborn data collection form

This form was used to assess procedures performed during the first and second stages of labor. In addition, it was used to assess the newborn’s condition after delivery. This form, created by researchers based on the literature, consisted of three sections. The first section contained questions about procedures such as dilation, effacement, amniotomy, etc. The second section included questions about perineal protection, pushing techniques, etc. during the second stage. The third section included questions about the height, weight, and APGAR score of the newborn. In total, these forms contained 33 questions 1 4 5 8 11 12 13 15 16 17 18 .

### Visual analog scale (VAS)

The VAS is a one-dimensional scale that can be used to assess the intensity of perceived pain. A rating of “0” indicates “no pain” and “10” indicates “the most severe pain.”

Trauma assessment and hemorrhage and length of episiotomy measurement form

This was used to collect data on trauma to the perineal area and hemorrhage quantity. Created by the researchers, it includes 13 questions assessing factors such as episiotomy, the type and degree of trauma, and hemorrhage volume. A postpartum hemorrhage follow-up collector bag (PBFCB) was used to measure the volume of the postpartum hemorrhage.

### Ethical considerations

Ethical approval (Decision number: 90057706-514.10 - 2018/13) was given by a Clinical Research Ethics Committee in Türkiye, on 18.04.2018. Written consent was given, and the consent forms were securely stored.

### Data analysis

The statistical analysis was done using SPSS version 23.0 (IBM Corp., Armonk, NY, USA). The significance level was accepted as *p* <.05. Descriptive statistics were demonstrated as numbers and percentages. For continuous variables, standard deviation, median, min., and max. were presented. The Kolmogorov–Smirnov test was used to assess whether data followed a normal distribution. Based on results, a one-way ANOVA was used to compare mean scores among more than two independent groups. Subsequently, Tukey’s test was used for identifying which groups differed. The Kruskal–Wallis test was used to determine differences among more than two independent groups. The Wilcoxon test was used to compare two dependent groups. The Friedman test was used to assess differences between more than two dependent groups and a chi-square test was used to analyze the relationship between two independent variables. In addition, Fisher’s exact test was used for n×m tables. The Pearson coefficient test was used to measure the association between two continuous variables.

## Results

### Homogeneity test and distribution of socio-demographic and obstetric characteristics in control and intervention groups

This assessment was conducted to present equivalence between four groups. There is no significance in age ( *p* =0.270), residence ( *p* =0.901), educational status ( *p* =0.566), or obstetric history, including week of gestation ( *p* =0.104), miscarriage/abortion ( *p* =0.465), and pregnancy intention ( *p* =0.191) ( Table 1 ).

**Table:** **Table 1** Homogeneity and distribution of socio-demographic characteristics in the control and intervention groups.

Variable	PM (n=30) Mean±SD		PWC (n=30) Mean±SD		PM+PWC (n=30) Mean±SD		Control (n=30) Mean±SD		Total (n=120) Mean±SD		Statistical Analysis	
											F	*p*
**Age**	26.43	±3.90	25.4	3±4.02	25.43	±4.02	24.57	±3.81	25.4	7±3.94	1.125	0.342
	**n**	**%**	**n**	**%**	**n**	**%**	**n**	**%**	**n**	**%**	**X** ^**2**^	***p***
**Education Level**												
Elementary School	4	13.3	2	6.7	6	20.0	4	13.3	16	13.3		
High School	9	30.0	13	43.3	8	26.7	11	36.7	41	34.2	7.730	0.566
Associate Degree	3	10.0	4	13.3	8	26.7	4	13.3	19	15.8		
Bachelor's Degree	14	46.7	11	36.7	8	26.7	11	36.7	44	36.7		
**Employment Status**												
Working	9	30.0	9	30.0	6	20.0	5	16.7	29	24.2	2.319	0.566
Not Working	21	70.0	21	70.0	24	80.0	25	83.3	91	75.8		
**Residence**												
City	28	93.3	29	96.7	30	100.0	29	96.7	26	96.7	2.062	0.901
District	2	6.7	1	3.3	–	–	1	3.3	4	3.3		
**Body Mass Index**												
Underweight	–	–	–	–	1	3.3	–	–	1	0.8		
Normal	9	30.0	15	50.0	2	6.7	4	13.3	19	15.8	10.859	0.232
Overweight	12	40.0	11	36.7	19	63.3	19	63.3	65	54.2		
Obese	9	30.0	30	100.0	8	26.7	7	23.3	35	29.2		
**Pregnancy Week**												
<40 weeks	14	46.7	21	70.0	12	40.0	17	56.7	64	53.3	6.161	0.104
40 weeks and>	16	53.3	9	30.0	18	60.0	13	43.3	56	46.7		
**Miscarriage/Abortion**												
Yes	5	16.7	1	3.3	3	10.0	4	13.3	13	10.8	3.112	0.465
No	25	83.3	29	96.7	27	90.0	26	86.7	107	89.2		
**Weight Gain During Pregnancy**												
8 kg and below	3	10.0	1	3.3	3	10.0	3	10.0	10	8.3	6.355	0.380
9–12	14	46.7	8	26.7	9	30.0	7	23.3	38	31.7		
13 kg and above	13	43.3	21	70.0	18	60.0	20	66.7	72	60.0		
**Pregnancy Intention**												
Intentional	27	90.0	29	96.7	30	100.0	30	100.0	116	96.7	4.397	0.191
Unintentional	3	10.0	1	3.3	–	–	–	–	4	3.3		

PM=perineal massage, PWC=perineal warm compress, PM+PWC=combined perineal massage and warm compress F=one-way analysis of variance (ANOVA), χ2=chi-square and Fisher’s exact test

### Primary outcomes: perineal trauma and degree of trauma

The effectiveness of massage and warm compresses to decrease perineal trauma in pregnant women was investigated across the groups ( Table 2 ). The perineal trauma rate was 6.7% in the PM group and 3.3% in the PWC group. This percentage was 10% in the PM+PWC group and 60% for the control group. Trauma incidence in the control group was significantly greater than the intervention groups. A statistically significant difference was found (chi-square test=40.417, *p* <0.001). Additionally, the degree of perineal trauma was assessed, and it was understood there was no trauma to participants exceeding the second degree. There was no first-degree trauma in the warm compress group. First-degree trauma was observed at a rate of 3.3% in the massage and massage+warm compress groups and 26.7% in the control group. When the difference between groups regarding first-degree trauma was examined, it was determined that there was a statistical significance ( *p* <0.05).

**Table:** **Table 2** Distribution of perineal trauma incidence in the control and intervention groups.

	PM (n=30) Mean±SD		PWC (n=30) Mean±SD		PM+PWC (n=30) Mean±SD		Control (n=30) Mean±SD		Total (n=120) Mean±SD		Statistical Analysis	
											F	*p*
	n	%	n	%	n	%	n	%	n	%	X ^2^	*p*
**Perineal Trauma**												
Yes	2 ^b^	6.7	1 ^b^	3.3	3 ^b^	10.0	18 ^a^	60.0	24	20.0	40.417	**<0.001** ^*^
No	28 ^b^	93.3	29 ^b^	96.7	27 ^b^	90.0	12 ^b^	40.0	96	80.0		
**Type of Perineal Trauma**												
Laceration	1	50.0	1	100.0	1	33.3	4	22.2	7	29.2	4.207	0.889
Perineal Tear	1	50.0	0	–	1	33.3	8	44.4	10	41.7		
Hematoma	0	–	0	–	1	33.3	6	33.3	7	29.2		
**Degree of Perineal Trauma**												
No Perineal Tear	29 ^a,b^	96.7	30 ^b^	100.0	29 ^a,b^	96.7	22 ^a^	73.3	110	91.7	13.386	**0.001** ^*^
1st Degree	1 ^a,b^	3.3	– ^b^	–	1 ^a,b^	3.3	8 ^a^	26.7	10	8.3		

* *p* <0.001, x ^2^ =chi-square and Fisher’s exact test, a-b=There is a difference between the two groups; a-a/b-b=There is no difference between the two groups.

### Secondary outcomes

The results of the measurement of postpartum hemorrhage were 221.66 ml (±85.78) in the massage group, 213.33 ml (±109.01) in the warm compress group, and 240.0 ml (±103.72) in the massage+warm compress group ( Table 3 ). In the control group, the incidence of postpartum hemorrhage was 410.83 (±211.48). The volume of postpartum hemorrhage in the control group was significantly greater than the intervention groups. A statistically significant difference was found between the groups (Kruskal-Wallis test: 25.158, *p* <0.001). Episiotomy lengths were 3.1 cm (±0.96) in the massage group, 2.63 cm (±0.94) in the warm compress group, and 2.98 cm (±0.88) in the massage+warm compress group. In the control group, it was measured as 4.13 (±0.88). The episiotomy length in the control group was greater than the intervention groups ( Table 3 ). A statistically significant difference was found between the groups in terms of episiotomy length (Kruskal-Wallis test: 36.434, *p* <0.001).

**Table:** **Table 3** Distribution of postpartum bleeding amount and episiotomy length in the control and intervention groups.

	Postpartum Bleeding Amount (ml)	Episiotomy Length (cm)
	Median (Min–Max)	Median (Min–Max)
**PM** (n=30)	221.66 (±85.78)	3.10 (±0.96)
**PWC** (n=30)	213.33 (±109.01)	2.63 (±0.94)
**PM+PWC** (n=30)	240.0 (±103.72)	2.98 (±0.88)
**Control** (n=30)	410.83 (±211.48)	4.13 (±0.88)
**x** ^**2**^ **;** ***p***	25.158;< 0.001*	36.434;< 0.001*
**Difference**	4–1,2,3	4–1,2,3

* *p* <0.001, x ^2^ =Kruskal-Wallis test, 1=PM, 2=PWC, 3=PM+PWC, 4=Control Group

In all groups, pain severity scores were evaluated when dilatation of the cervix was 3–4 cm, 5–7 cm, or 8–10 cm. In addition, pain scores were compared before and after application in the intervention groups. When dilatation of the cervix was 5–7 cm and 8–10 cm, pain scores were greater in the control group than in the PW and PWC groups ( *p* <0.05). Furthermore, a significant correlation was found between the pain score before intervention and after three interventions in the PW group ( Table 4 ). It has been determined that perineal trauma is reduced after each application of a warm compress. A statistically significant difference was detected ( *p* <0.05).

**Table:** **Table 4** Distribution of pain scores in the control and intervention groups.

	Intervention 1 (Dilation 3–4 cm)		Statistical Analysis	Intervention 2 (Dilation 5–7 cm)		Statistical Analysis	Intervention 3 (Dilation 8–10 cm)		Statistical Analysis
	Before Mean±SD	After Mean±SD		Before Mean±SD	After Mean±SD		Before Mean±SD	After Mean±SD	
**PM** (n=30)	4.17±1.53	4.40±1.90	t=–1.126	7.0±1.48	6.93±1.89	t=–0.226	9.03±0.96	9.0±0.98	t=–1.00
			*p* =0.269			*p* =0.823			*p* =0.326
**PWC** (n=30)	4.40±1.86	4.27±1.83	t=–2.112	6.70±1.57	6.37±1.65	^t=3.808^	8.80±1.03	8.50±1.16	t=–3.071
			***p*** **=0.043** ^*^			***p*** **=0.001** ^**^			**p=0.005** ^**^
**PM+PWC** (n=30)	3.33±1.02	3.43±1.10	t=–0.722	6.33±0.95	6.13±1.25	t=1.439	8.97±0.99	8.93±1.04	t=0.571
			*p* =0.476			*p* >0.999			*p* =0.573
**Control** (n=30)	–	4.37±2.26	–	–	7.70±1.80	–	–	9.73±0.82	–
**Statistical Analysis (x** ^**2**^ **;** ***p*** **)**	F=4.103	F=1.893		F=1.785	F=5.246		F=0.435	F=7.640	
	*p* =0.20	*p* =0.135		*p* =0.174	***p*** **=0.002** ^*^		*p* =0.649	***p*** **<0.001** ^*^	
**Difference Between Groups**					4–2,3			4–2,3	

* *p* <0.05, **p<0.01, 1=PM, 2=PWC, 3=PM+PWC, 4=control group, t=paired-samples t test, F=one-way ANOVA

## Discussion

Massage and warm compress methods are nonpharmacological methods recommended for a better childbirth result and to reduce trauma to the perineum, pain around the perineal area, hemorrhage and episiotomy rates, and to increase the flexibility of perineum 4 5 19 . In this study, the effectiveness of massage and warm compresses on reducing trauma and pain were investigated. Additionally, the volume of postpartum hemorrhage and the length of the episiotomy were measured.

Our study revealed that massage and warm compress applications reduced perineal trauma. In a study that shows the result of massage on the perineum, trauma incidence was 63.2% in the massage group and 60.5% in the control group 20 . Another study that investigated the effects of Vaseline massage revealed that trauma incidence was 73.3% in the intervention group and 96.0% in the control group 15 . In a study evaluating the effectiveness of perineal warm compresses, the rate of perineal trauma was 73.0% in the compress group and 93.3% in the control group 12 .

Considering complications and unwanted effects of perineal trauma, the severity of perineal trauma is as important as the degree of trauma. Some studies have shown that massage and warm compresses reduce the severity of perineal trauma 4 5 16 . In contrast to the literature, the absence of a second and higher degree of perineal trauma in our research may be attributed to the effective management of labor and application of correct clinical procedures. Our study further revealed that the combined use of massage and warm compresses did not provide an advantage. For this reason, it is thought that any of these methods alone will be sufficient when application time, workforce, and cost of the healthcare team are considered.

According to the literature, the volume of hemorrhage increases due to routine episiotomy and perineal trauma, accounting for approximately 20.0% of cases of postpartum hemorrhage. For this reason, it is important to follow up hemorrhage with objective measurement tools in terms of early and effective intervention in postpartum hemorrhage 9 21 . Preterm postpartum hemorrhage is also associated with episiotomy. Since routine episiotomy is common, especially in primiparous patients, perineum-preserving approaches are recommended to decrease the risk of trauma to the perineum, hemorrhage, and episiotomy length 16 21 22 23 . In line with our research results, perineal massage and a warm compress did not change the physicians’ preference for episiotomy. However, evaluating the flexibility of the perineum was preferable for a shorter episiotomy. Thus, it is thought that there is a decrease in the volume of postpartum hemorrhage due to the reduction in episiotomy length and trauma.

Perineal trauma is frequently experienced during the second stage of labor. In this period, trauma occurs due to the tension created by the fetal head in the perineal tissue. Perineal trauma is associated with the implementation of an episiotomy and type of suturing 17 24 . It has been reported that approaches such as warm compress application and massage are effective for decreasing perineal trauma. Different studies reported that perineal massage is effective in reducing perineal trauma 12 19 . Moreover, there are studies that have shown that warm compresses are effective in decreasing perineal trauma 2 14 16 25 . The findings in the literature and our findings coincide. Perineal trauma seems to be reduced in relation to relaxation, especially in the tissues, as a result of a warm compress.

### Limitations

First, there is no field-specific scale for evaluating perineal pain and trauma. Second, our findings cannot be generalized to different groups because this study included a single center only, and the researcher who conducted the study was not blinded.

## Conclusion

The warm compress intervention and massage application methods are effective for decreasing trauma to the perineum, pain, hemorrhage volume, and episiotomy length. With these methods performed during labor, it is possible to obtain perineal flexibility and to perform a shorter episiotomy. It is thought that there is a decrease in the volume of postpartum hemorrhage due to the reduction in episiotomy length and trauma. In addition, warm compresses are particularly effective in reducing perineal pain, as there is a relaxation of tissues. However, the use of two methods together does not offer an advantage. These techniques can be used independently of each other and they are recommended to decrease the rate of perineal trauma, hemorrhage, pain, and episiotomy length.
